# Factors linked to poor self-rated health in thyroid disorder patients: findings from LASI Wave-I

**DOI:** 10.1186/s13044-025-00229-8

**Published:** 2025-05-13

**Authors:** Pawan Kumar, Arunima Sen, Priyanshu Priyanshu, Mahalaqua Nazli Khatib, R. Roopashree, Mandeep Kaur, Manish Srivastava, Amit Barwal, G. V. Siva Prasad, Pranchal Rajput, Muhammed Shabil, Rukshar Syed, Gajendra Sharma, Abhay M. Gaidhane, Diptismita Jena, Ganesh Bushi, Rachana Mehta, Amit Verma, Hashem Abu Serhan, Ahmad Neyazi, Prakasini Satapathy

**Affiliations:** 1 Department of Community Medicine, School of Public Health, Postgraduate Institute of Medical Education and Research, Chandigarh, India; 2 Global Center for Evidence Synthesis, Chandigarh, India; 3 Division of Evidence Synthesis, Global Consortium of Public Health and Research, Datta Meghe Institute of Higher Education, Wardha, Maharashtra India; 4 Department of Chemistry and Biochemistry, School of Sciences, JAIN (Deemed to be University), Bangalore, Karnataka India; 5 Department of Allied Healthcare and Sciences, Vivekananda Global University, Jaipur, Rajasthan, 303012 India; 6 Department of Endocrinology, NIMS University, Jaipur, Rajasthan India; 7 Chandigarh Pharmacy College, Chandigarh Group of College, Jhanjeri, Mohali, 140307 Punjab India; 8 Department of Chemistry, Raghu Engineering College, Visakhapatnam, 531162 Andhra Pradesh India; 9 School of Applied and Life Sciences, Division of Research and Innovation, Uttaranchal University, Dehradun, Uttarakhand India; 10 University Center for Research and Development, Chandigarh University, Mohali, Punjab India; 11 Medical Laboratories Techniques Department, AL-Mustaqbal University, Hillah, Babil, 51001 Iraq; 12 IES Institute of Pharmacy, IES University, Bhopal, 462044 Madhya Pradesh India; 13 New Delhi Institute of Management, Delhi, India; 14 Global Health Academy, School of Epidemiology and Public Health, Jawaharlal Nehru Medical College, Datta Meghe Institute of Higher Education, Wardha, Maharashtra India; 15 Center for Global Health Research, Saveetha Institute of Medical and Technical Sciences, Saveetha Medical College and Hospital, Saveetha University, Chennai, Tamil Nadu India; 16 School of Pharmaceutical Sciences, Lovely Professional University, Phagwara, Punjab India; 17 University of Cyberjaya, Persiaran Bestari, Cyber 11, Selangor Darul Ehsan, Cyberjaya, 63000 Malaysia; 18 Clinical Microbiology, RDC, Manav Rachna International Institute of Research and Studies, Faridabad, 121004 Haryana India; 19 Department of Medicine, Graphic Era Institute of Medical Sciences, Graphic Era (Deemed to be University, Clement Town, Dehradun, Uttarakhand India; 20 Department of Ophthalmology, Hamad Medical Corporation, Doha, Qatar; 21 Afghanistan Center for Epidemiological Studies, Herat, Afghanistan; 22 Faculty of Medicine, Ghalib University, Herat, Afghanistan; 23 Centre of Research Impact and Outcome, Chitkara University, Rajpura, 140417 Punjab India; 24 Faculty of Data Science and Information Technology, INTI International University, Nilai, Malaysia

**Keywords:** Hashimoto's Thyroiditis, Hypothyroidism, Hyperthyroidism, Healthcare utilization, thyroid cancer, Goitre, Good Health and Well-being

## Abstract

**Background:**

Thyroid disorders affect the physical, behavioural, and psychological aspects of an individual, leading to poor self-rated health (SRH). Hence, we aimed to determine the prevalence of poor SRH and the factors associated with it among thyroid disorder patients.

**Methods:**

This is an observational study consisting of 2336 thyroid disorder patients from LASI, 2017-19. Descriptive statistics were employed to calculate prevalence. The association between poor SRH and socio-demographic variables was evaluated using regression analysis, with results expressed as (AOR) and 95% CI.

**Results:**

The findings showed poor self-rated health predictors among thyroid disorder patients, where 25% rated their health as poor. Significant predictors included older age, with patients aged ≥ 75 years having a higher likelihood of reporting poor health (aOR = 2.36, 95% CI = 1.32–4.22, *p* = 0.004), and rural residence (aOR = 1.34, 95% CI = 1.07–1.67, *p* = 0.011). Belonging to the OBC caste (aOR = 1.57, 95% CI = 1.23–2.00, *p* < 0.001) and practicing Christianity (aOR = 1.90, 95% CI = 1.25–2.89, *p* = 0.003) were also associated with increased odds of poor SRH. Previous employment (aOR = 1.65, 95% CI = 1.20–2.25, *p* = 0.002), co-morbidities (aOR = 2.59, 95% CI = 1.88–3.59, *p* < 0.001), and lower education levels (aOR = 1.50, 95% CI = 1.06–2.13, *p* = 0.022) were significant. Limitations in activities of daily living and instrumental activities of daily living were linked to poorer health outcomes (aOR = 1.76, 95% CI = 1.33–2.31, *p* < 0.001; IADL: aOR = 1.41, 95% CI = 1.11–1.79, *p* = 0.004). Depression (aOR = 1.84, 95% CI = 1.32–2.56, *p* < 0.001) and healthcare utilization in the past year (aOR = 1.86, 95% CI = 1.33–2.58, *p* < 0.001) also predicted poor SRH, with most healthcare utilization (79.8%) occurring in private facilities.

**Conclusion:**

The study highlights a high prevalence of poor SRH among patients, with significant associations observed with age, residence, comorbidity, and healthcare utilization. Targeted interventions focusing on healthcare access, physical activity, and mental health support are crucial to improve SRH.

## Introduction

Thyroid disorders encompass a range of conditions affecting the thyroid gland, which can be categorized based on clinical severity, serum hormone levels, the presence of thyroid antibodies, or their effects on target tissues. These conditions comprise hypothyroidism, hyperthyroidism, thyroiditis, thyroid tumours, and thyroid cancer [1, 2]. In iodine-sufficient countries, hypothyroidism affects 1–2% of people, with a higher prevalence among older women [3]. Prevalence of overt hyperthyroidism ranges from 0.2 to 1.3%. Both conditions are more common in women [2]. In iodine replete populations, while Graves’ disease is the leading cause of hyperthyroidism, Iodine deficiency and autoimmune disease such as Hashimoto thyroiditis account for the vast majority of primary hypothyroidism cases [3]. Thyroid disorders can significantly impact metabolic, cardiovascular, and psychological health, often influencing overall health-related quality of life (HRQoL) and self-rated health (SRH) [4].

Thyroid dysfunction ranges from overt syndromes to subtle subclinical disturbances, affecting physical and mental health and influencing SRH [4]. Hypothyroidism has been directly linked to reduced HRQoL due to its symptoms such as fatigue, depression, and cognitive sluggishness, which can impair daily functioning and life satisfaction. Various studies highlight that thyroid diseases affect not only the physical realm but also emotional and social well-being [5, 6]. Patients with thyroid disorders often experience a significant impact on their work capability and social interactions due to fluctuating energy levels and mood disturbances [7]. This multidimensional impact can lead to increased sick leaves, reduced work productivity, and even unemployment or disability, further deteriorating the QoL [5].

Despite effective treatment, many thyroid disorder patients continue to experience persistent symptoms. Inadequate strategies, such as using levothyroxine alone, may not restore euthyroid status, particularly in those with certain polymorphisms [8, 9]. This persistence underscores the need for personalized medical approaches and contributes to poor SRH. Demographic factors like age, gender, and socio-economic status also significantly influence SRH in thyroid disorder patients [10]. Women are more susceptible to thyroid dysfunction and its complications during life stages like pregnancy and menopause, further impacting their health perception [3, 11].

India’s transition from iodine-deficient to iodine-replete status comes with new challenges. There is increasing evidence that excess iodine exposure from iodine supplementation potentially trigger autoimmune thyroid dysfunction especially among susceptible populations like developing foetuses, neonates, the elderly, and those with pre-existing thyroid disease [12, 13]. The National Family Health Survey (NFHS) reported an increase in self-reported goitre or thyroid disorder prevalence from 2.2% in 2015–2016 to 2.9% in 2019–2021, with higher rates in females and an increasing trend with age [14]. By understanding the determinants of poor self-rated health among thyroid disorder patients, targeted interventions can be developed to improve quality of life and health outcomes. Insights from the data can help refine therapeutic strategies and healthcare policies tailored to this population, enhancing their overall well-being. Continuous monitoring and evaluation are vital for adapting these strategies to meet evolving health needs. This study aims to determine the prevalence of poor SRH and the factors associated with it among thyroid disorder patients using data from the Longitudinal Aging Study in India (LASI) Wave 1, 2017-19.

## Methods

### Data source

This study utilized data from the Longitudinal Aging Survey of India (LASI) Wave 1 for the years 2017-19. The survey was a collaborative effort undertaken by Harvard T.H. Chan School of Public Health, the International Institute for Population Sciences (IIPS), and the University of Southern California, employing a multistage stratified area probability cluster sampling methodology. The unit of observation was defined as “LASI Eligible Households” (LEH), which included households with at least one member aged 45 years and above. Conducted across all 36 Indian states and union territories, the first wave of LASI covered a sample of 73,408 individuals who were 45 years and older along with their spouses regardless of their age, using a three-stage sampling method in rural areas and a four-stage method in urban areas. The selected households received an individual survey questionnaire. The individual survey collected information on demographics, self-reported health status, chronic and endemic diseases, and healthcare utilization. The methodology used during the first wave of LASI is detailed on the IIPS, Mumbai’s official website [15–17]. This study comprises 2,336 respondents who consented to report on thyroid disorders, making them eligible for the current analysis.

### Variables

#### Outcome variable

##### Self-rated health among patients with thyroid disorders

Self-rated health (SRH) was assessed by asking respondents, “How would you rate your overall health?” with the response options “Very poor”, “Poor”, “Fair”, “Good”, and “Very good”. For analysis, SRH was categorized into two groups: ‘good’, encompassing “Very good”, “Good”, and “Fair”; and ‘poor’, including “Poor” and “Very poor”. To enable a clearer interpretation, a binary variable was constructed, assigning a value of 1 to “good SRH for thyroid disorders” and a value of 2 to “poor SRH for thyroid disorders”.

#### Independent variables

##### Socio-demographics

The following variables were included in the analysis - Age groups (18–44 years, 45–59 years, 60–74 years, > 75 years), Sex (Male, Female), Residence (Rural, Urban), Region (North, West, Central, East, North-east, and South), Religion (Hindu, Muslim, Christian, and others), and Caste (Scheduled Caste (SC), Scheduled Tribe (ST), Other Backward Classes (OBCs), and none). Education was recategorized into no education/below primary, Primary/Middle school, Matric/Secondary, and higher secondary and above. Furthermore, Monthly per capita expenditure (MPCE) was categorized as Poor, Middle, or Rich. MPCE was calculated by dividing total household consumption expenditure by the number of household members, including spending on food, non-food items, health, education, and utilities. IIPS imputed data for consumption was used. Marital status was recategorized as having a partner or not.

##### Chronic illnesses

Comorbidity, which are self-reported in LASI, was defined as the coexistence of any other chronic physical health conditions in the same individual apart from thyroid disorder. A composite indicator was developed, coded as 1 for comorbidity present and 0 for comorbidity absent. Based on an extensive literature review, the following diseases were included in the analysis: Hypertension/High blood pressure, Diabetes or High blood sugar, Cancer or Malignant tumor, Chronic lung disease, Chronic heart diseases, Stroke, Arthritis or rheumatism, any neurological or psychiatric problems (depression, Alzheimer’s/Dementia, unipolar/bipolar disorders, convulsions, Parkinson’s etc.), High Cholesterol, and other chronic conditions such as Gastro-intestinal problems, and Skin diseases [18, 19]. 

##### Behavioural characteristics and physical activity

Behavioural characteristics included current smoking, current use of smokeless tobacco, use of alcohol in the last 3 months, physical activity, and practicing yoga. Physical activity status was assessed through a question regarding participation in sports or vigorous activities or moderately energetic activities such as running or jogging, swimming, going to a health centre or gym, cycling, or engaging in activities like digging with a spade or shovel, heavy lifting, chopping, farm work, fast bicycling, cycling with loads, or participating in cleaning house, washing clothes by hand, fetching water or wood, drawing water from a well, gardening, bicycling at a regular pace, walking at a moderate pace, dancing, floor, or stretching exercises. Responses were recoded as ‘yes’ (every day, more than once a week) or ‘no’ (once a week, one to three times a month, or hardly ever or never). According to WHO norms, individuals engaged in moderate physical activity (at least 150 min throughout the week) or vigorous physical activity (at least 75 min throughout the week). Therefore, if either vigorous or moderate activity was yes, the participant was physically active.

##### Assessment of daily living activities, depression, and healthcare utilization

Limitations in Activities of Daily Living (ADL) were assessed by questioning respondents about difficulties lasting more than 3 months with dressing, walking across the room, bathing, eating, getting in or out of bed, or using the toilet. Instrumental Activities of Daily Living (IADL) were assessed by asking about difficulties expected to last for at least 3 months, such as shopping for groceries, preparing a hot meal, making a telephone call, managing money, and getting around in unfamiliar places.

Depression was calculated using the Composite International Diagnostic Interview - Short Form (CIDI-SF) on a scale of 0–10. This scale estimates a probable psychiatric diagnosis of major depression and has been widely used in population-based health surveys Responses, except for items “2” and “3”, were in binary format (0 for “No” and 1 for “Yes”). Individuals indicating feeling sad, blue, or depressed “all day long” or “most of the day” were coded as “Yes”; similarly, those feeling so “every day” or “almost every day” were also coded as “Yes”. Scores were summed to create the CIDI-SF depression scale (0–10). Indian older adults with a score of 5 or more were categorized as “Depressed”; those with 4 or less were classified as “Not depressed”.

Healthcare utilization in the past 12 months was assessed using the question: “In the past 12 months, have you visited any healthcare facility or has any health professional visited you?” This encompassed visits to public facilities (such as health post/sub-centers, primary health center/urban health centre, and government hospitals), private facilities (private hospitals, clinics, and AYUSH hospitals), and other facilities (health camps, mobile healthcare units, and pharmacies). Multiple answers were accepted, and all facilities attended by a single participant in the past year were recorded. Health care providers were categorized into various types, including doctors, dentists, nurses/midwives, physiotherapists, pharmacists, and traditional/folk healers. Reasons for visits and reasons for not seeking care were also recorded.

### Statistical analysis

Data was analysed using SPSS trial version. Descriptive analysis was performed to describe the study sample. For each study variable, unweighted frequency and weighted percentages were calculated. Binary logistic regression analyses were performed to establish the association between the outcome variables and explanatory variables. Multivariable logistic regression was used to determine the adjusted effect of predictor variables on the likelihood of poor-SRH. Factors found significant on bivariate analysis were included in multivariable regression. A *P*-value of < 0.05 was considered to be statistically significant.

### Ethical considerations

This research employed data obtained ethically from IIPS, Mumbai, with full authorization, using secondary and anonymized LASI wave-1 data, ensuring no participant risk and appropriate citation.

## Results

The study population comprised 2,336 individuals diagnosed with thyroid disorders with 25.1% reporting a poor self-rated health. The sample was predominantly female (83.1%), and almost half (50.4%) were in the 45–59 years age group. Geographically, a higher number of participants were from Southern states (31.7%), and more than half resided in urban areas (59.2%). Around 42% belonged to Other Backward Classes (OBC), and 36.6% reported no caste designation. The religious composition was predominantly Hindu (76.7%), followed by Muslim (16.2%). Marital status revealed that 79.4% had a partner, while 20.6% did not. Regarding employment, 45.5% of the participants had never worked. Multimorbidity was prevalent, with 77.7% of participants having at least one other chronic condition besides thyroid disorder. Almost 44% were in the rich category based on their mean per capita expenditure (MCPE), and 41% had no education or less than primary education. Behavioral characteristics showed that 3.9% were current smokers, 11.0% used smokeless tobacco, and 1.8% had consumed alcohol in the last three months. Physical activity was reported by 71.2% of participants, and yoga practice was noted in 14.5%. Limitations in ADL were reported by 22.2% and limitations in IADL by 38.6%. Depression, assessed using the CIDI-SF tool, was found in 9.6% of participants. [Table 1 shows Socio-demographic and behavioral characteristics of patients with thyroid disorders in India, LASI wave − 1, 2017–2019].

**Table 1:** Socio-demographic and behavioral characteristics of patients with thyroid disorders in India, LASI wave -1, 2017-2019 (*N*=2336)

Variable	*n*	(%)*
**Age category in years**		
18– 44	364	(16.0)
35– 59	1214	(50.4)
60– 74	634	(28.0)
*>* 75	124	(5.6)
**Geographical region**		
North	555	(13.1)
Central	160	(13.9)
East	427	(24.3)
Northeast	172	(2.5)
West	296	(14.5)
South	726	(31.7)
**Place of residence**		
Rural	916	(40.8)
Urban	1420	(59.2)
**Sex**		
Male	379	(16.9)
Female	1957	(83.1)
**Caste**		
Scheduled caste	280	(12.8)
Scheduled tribe	186	(3.9)
Other backward class (OBC)	780	(42.4)
None	958	(36.6)
Missing	132	(4.3)
**Religion**		
Hinduism	1628	(76.7)
Islam	419	(16.2)
Christianity	176	(3.8)
None	3	(0.04)
Others	110	(3.3)
**Marital Status**		
No partner	495	(20.6)
Has partner	1841	(79.4)
**Work Status**		
Currently working	596	(28.8)
Previously worked	525	(25.7)
Never worked	1215	(45.5)
**Multimorbidity**		
No	519	(22.1)
Yes	1814	(77.7)
Missing	3	(0.2)
**MCPE category**		
Poor	586	(24.1)
Middle	632	(26.3)
Rich	933	(43.6)
Missing	185	(6.0)
**Education**		
No education/ Less than Primary	889	(40.9)
Primary/ Middle school	344	(14.4)
Matric/ Secondary	635	(24.2)
Higher secondary and above	468	(20.5)
**Smoking**,** current**		
No	2232	(96.1)
Yes	104	(3.9)
**Current use of smokeless tobacco**		
No	2122	(89.0)
Yes	214	(11.0)
**Alcohol use in last 3 months**		
No	2258	(97.2)
Yes	54	(1.8)
Missing	24	(1.0)
**Physical activity**		
No	698	(27.8)
Yes	1614	(71.2)
Missing	24	(1.0)
**Yoga**		
No	1938	(84.5)
Yes	374	(14.5)
Missing	24	(1.0)
**Limitations in ADL**		
No	1923	(77.4)
Yes	405	(22.2)
Missing	8	(0.4)
**Limitations in IADL**		
No	1526	(61.1)
Yes	802	(38.6)
Missing	8	(0.4)
**Depression**		
No	2091	(88.8)
Yes	208	(9.6)
Missing	37	(1.6)

MCPE– Mean per capita expenditure ADL– Activities of daily living IADL- Instrumental Activities of Daily Living * weight adjusted proportion

Multivariable regression analysis revealed several factors significantly associated with poor self-rated health (SRH) among thyroid disorder patients. Older age was a significant predictor, with the odds of poor SRH increasing with age: 45–59 years (aOR = 1.71, 95% CI = 1.18–2.47, *p* = 0.004), 60–74 years (aOR = 1.96, 95% CI = 1.31–2.94, *p* = 0.001), and > 75 years (aOR = 2.36, 95% CI = 1.32–4.22, *p* = 0.004). Rural residence was also associated with higher odds of poor SRH (aOR = 1.34, 95% CI = 1.07–1.67, *p* = 0.011). Caste and religion were significant predictors, with OBC participants (aOR = 1.57, 95% CI = 1.23-2.00, *p* < 0.001) and Christians (aOR = 1.90, 95% CI = 1.25–2.89, *p* = 0.003) more likely to report poor SRH [Table 2].

**Table 2:** Factors associated with poor self-reported health among patients with thyroid disorders in India, LASI wave -1, 2017-2019

Variable	uOR	(95% CI)		*P* value	aOR	(95% CI)		*P* value
**Age category in years**								
18– 44	1							
45– 59	2.11	(1.51-	2.95)	**< 0.001**	1.71	(1.18-	2.47)	**0.004**
60– 74	3.10	(2.18-	4.39)	**< 0.001**	1.96	(1.31-	2.94)	**0.001**
*>* 75	5.20	(3.22-	8.37)	**< 0.001**	2.36	(1.32-	4.22)	**0.004**
**Place of residence**								
Urban	1							
Rural	1.51	(1.25-	1.83)	**< 0.001**	1.34	(1.07-	1.67)	**0.011**
**Sex**								
Male	1							
Female	0.97	(0.75-	1.25)	0.82				
**Caste**								
None	1							
Scheduled caste	1.33	(0.98-	1.81)	0.06	1.26	(0.89-	1.77)	0.192
Scheduled tribe	0.89	(0.60-	1.32)	0.57	0.80	(0.50-	1.25)	0.324
Other backward class (OBC)	1.50	(1.21-	1.86)	**< 0.001**	1.57	(1.23-	2.00)	**<0.001**
**Religion**								
Hinduism	1							
Islam	1.02	(0.80-	1.31)	0.85	1.03	(0.76-	1.40)	0.833
Christianity	1.44	(1.03-	2.02)	**0.04**	1.90	(1.25-	2.89)	**0.003**
None	1.52	(0.14-	16.87)	0.73	2.41	(0.19-	30.68)	0.498
Others	0.81	(0.50-	1.30)	0.39	0.79	(0.48-	1.32)	0.372
**Marital Status**								
Has partner	1							
No partner	1.69	(1.36-	2.10)	**<0.001**	1.08	(0.83-	1.40)	0.579
**Work Status**								
Currently working	1							
Previously worked	2.48	(1.88-	3.26)	**< 0.001**	1.65	(1.20-	2.25)	**0.002**
Never worked	1.49	(1.17-	1.91)	**0.001**	1.29	(0.98-	1.71)	0.070
**Co-morbidity**								
No	1							
Yes	3.66	(2.71-	4.95)	**< 0.001**	2.59	(1.88-	3.59)	**<0.001**
Missing								
**MCPE category**								
Poor	1							
Middle	1.05	(0.81-	1.36)	0.70				
Rich	0.94	(0.74-	1.19)	0.59				
Missing								
**Education**								
Higher secondary and above	1							
No education/ Less than Primary	2.54	(1.89-	3.40)	**< 0.001**	1.50	(1.06-	2.13)	**0.022**
Primary/ Middle school	2.12	(1.49-	3.00)	**< 0.001**	1.33	(0.89-	1.99)	0.158
Matric/ Secondary	1.89	(1.38-	2.58)	**< 0.001**	1.62	(1.15-	2.30)	**0.006**
**Smoking**,** current**								
No	1							
Yes	1.17	(0.75-	1.81)	0.49				
**Current use of smokeless tobacco**								
No								
Yes	1.29	(0.95-	1.76)	0.11				
**Alcohol use in last 3 months**								
No	1							
Yes	0.67	(0.33-	1.33)	0.25				
**Physical activity**								
Yes	1							
No	1.75	(1.44-	2.13)	**< 0.001**	1.31	(1.04-	1.64)	**0.021**
**Yoga**								
Yes	1							
No	1.18	(0.91-	1.53)	0.22				
**Limitations in ADL**								
No	1							
Yes	2.74	(2.18-	3.43)	**< 0.001**	1.76	(1.33-	2.31)	**<0.001**
**Limitations in IADL**								
No	1							
Yes	2.38	(1.96-	2.88)	**< 0.001**	1.41	(1.11-	1.79)	**0.004**
**Depression**								
No	1							
Yes	2.42	(1.81-	3.25)	**<0.001**	1.84	(1.32-	2.56)	
**Healthcare utilization in last 12 months**								
No	1							
Yes	2.12	1.57	2.85	**<0.001**	1.86	(1.33-	2.58)	**<0.001**

ADL– Activities of daily living IADL- Instrumental Activities of Daily Living

Work status influenced SRH, with previously employed individuals having higher odds of poor SRH (aOR = 1.65, 95% CI = 1.20–2.25, *p* = 0.002). Comorbidity significantly increased the likelihood of poor SRH (aOR = 2.59, 95% CI = 1.88–3.59, *p* < 0.001). Educational level was inversely related to poor SRH, with lower educational attainment associated with higher odds of poor SRH: no education/less than primary (aOR = 1.50, 95% CI = 1.06–2.13, *p* = 0.022) and matric/secondary education (aOR = 1.62, 95% CI = 1.15–2.30, *p* = 0.006) [Table 2].

Physical inactivity was linked to higher odds of poor SRH (aOR = 1.31, 95% CI = 1.04–1.64, *p* = 0.021). Limitations in ADL (aOR = 1.76, 95% CI = 1.33–2.31, *p* < 0.001) and IADL (aOR = 1.41, 95% CI = 1.11–1.79, *p* = 0.004) were significant predictors of poor SRH. Depression was strongly associated with poor SRH (aOR = 1.84, 95% CI = 1.32–2.56, *p* < 0.001). Healthcare utilization in the past 12 months also emerged as a significant factor, with those utilizing healthcare services showing higher odds of poor SRH (aOR = 1.86, 95% CI = 1.33–2.58, *p* < 0.001) [Table 2 shows Factors associated with poor self-reported health among patients with thyroid disorders in India, LASI wave − 1, 2017–2019].

Regarding healthcare utilization, 84.6% of participants had sought healthcare in the past 12 months. The reasons for seeking healthcare included preventive checkups (13.3%), regular treatment/checkup/routine follow-up (31.6%), and sickness (65.2%). Among those who did not seek healthcare, the primary reasons were not getting sick (57.3%) and illness not being serious (10.1%). Most of the healthcare utilization was in private facilities (79.8%), followed by government facilities (45.6%), and other facilities (15.2*%)* [Table 3 shows Healthcare utilization of patients with thyroid disorders in India, LASI wave − 1, 2017–2019].

**Table 3:** Healthcare utilization of patients with thyroid disorders in India, LASI wave -1, 2017-2019

Variable name	*n*		(%)
**Healthcare utilization in previous 12 months** (***N***= **2336**)			
No		386	(15.4)
Yes		1950	(84.6)
**Reason for seeking help** (***N***=**1950**)			
Preventive checkup		265	(13.3)
Regular treatment/checkup/routine follow		630	(31.6)
Sickness		1299	(65.2)
Injury/Violence		46	(2.3)
Others		15	(0.8)
**Reason for not seeking help** (***N***=**316**)			
Did not get sick		221	(57.3)
Illness was not serious		39	(10.1)
Had medicine at home		35	(9.1)
Not enough money or cost was too high		7	(1.8)
Others		14	(4.4)
**Healthcare facility type visited in past 12 months** (***N***=**1923**)*			
Private		1534	(79.8)
Government		876	(45.6)
Others		293	(15.2)

*Multiple options were allowed

Note: Others for reason for not seeking care include: Needed to work Didnt want to give up a days work 5 Treatment was unlikely to be effective 7 Nobody to accompany 8 No quality facilities available nearby 11 No healthcare facility nearby 12 Other, please specify HC005_other

Among participants who sought healthcare in the past 12 months, 1534 (79.8%) visited private facilities, 876 (45.6%) utilized government ones, and 293 (15.2%) visited other facilities such as health camps, mobile units, or home visits

**Fig. 1:**
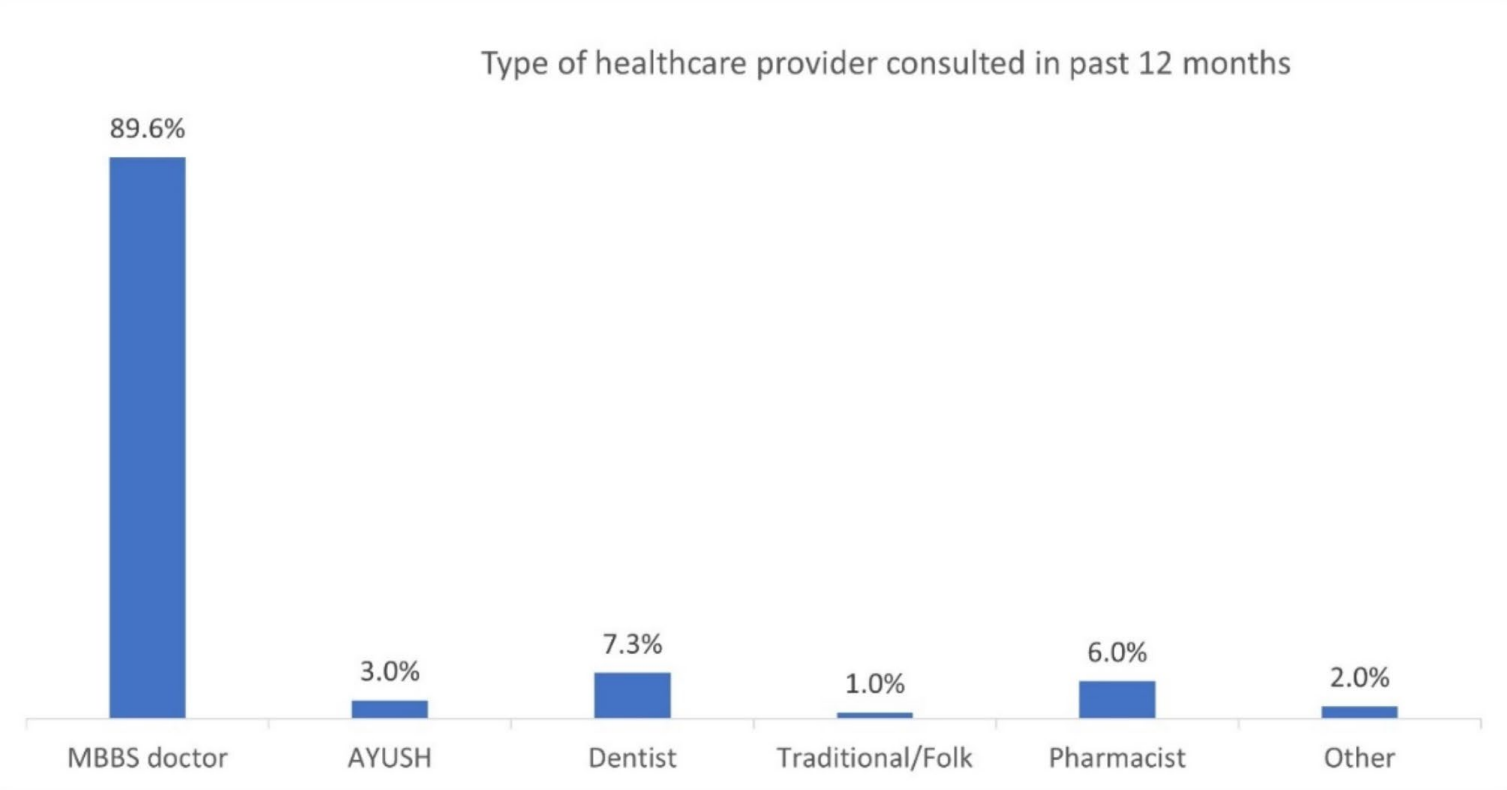
Type of healthcare provider consulted by patients with thyroid disorders, India, LASI wave 1, 2017-2019 (*N*=1910)

The figure on the type of healthcare provider consulted in the past 12 months showed that 89.6% of participants consulted MBBS doctors, 7.3% consulted dentists, 6.0% consulted pharmacists, 3.0% consulted AYUSH practitioners, 1.0% consulted traditional/folk healers, and 2.0% consulted other types of providers [Fig. 1 depicts Type of healthcare provider consulted by patients with thyroid disorders, India, LASI wave 1, 2017–2019].

## Discussion

The present study revealed that 1 in 4 (25.1%) individuals with thyroid disorders perceived their health as poor. Several key factors influencing SRH among thyroid patients were identified. Age, residence, caste, religion, employment status, comorbidity, education, physical activity, limitations in ADL, limitations in IADL, depression, and healthcare utilization were found to be significantly associated.

The odds of reporting poor SRH were notably higher among individuals aged 45 years and above compared to those in the 18–44 age group, with the highest odds observed in the 75 years and above age category. These findings are consistent with broader research demonstrating a decline in overall SRH as individuals age, particularly evident in the oldest-old age group [20–22]. An online survey of individuals with self-reported hypothyroidism also reported similar results. However, in that study, QoL declined with age and tended to be better in men than women [10]. Other studies examining the effect of gender on self-rated health or quality of life have reported similar findings [23]. However, our findings indicated that gender did not significantly influence self-rated health. The high prevalence of comorbidities in our study population may have contributed to the lack of significant gender differences.

Regarding urban-rural disparities in SRH, studies in India utilizing data from national surveys such as the National Sample Survey (NSS) and Building a Knowledge Base on Population Ageing in India (BKPAI) suggest that rural populations report poorer SRH [10, 20, 22]. In our study, it was observed that the situation was no different among thyroid patients. Rural residents had higher odds of rating their SRH as poor compared to urban residents. These findings likely stem from the advantageous position of urban areas in terms of the availability and accessibility of health services. Similar findings were reported by other studies conducted in India and abroad [18, 24]. A study conducted among residents of USA reported that, remote rural counties had the greatest odds of reporting bad health when compared with metropolitan residents due to structural disadvantage [24]. 

In this study, individuals with thyroid disorders who belonged to the Other Backward Class (OBC) and the Christian community exhibited higher odds of reporting poor SRH. In India, factors such as poverty, social exclusion, and spatial injustice are more prevalent among the marginalised communities, potentially impacting their SRH negatively [25]. Other studies from India have reported similar findings [26, 27]. In the current study, individuals belonging to the SC/ST communities did not exhibit a significant difference in SRH compared to those without a caste designation. A study, which investigated whether decadal differences in poor SRH could be attributed to the socio-economic context of older adults, found that the relationship between poor SRH and the SC/ST population discontinued in 2014, suggesting the efficacy of government targeted intervention programs for the SC/ST communities [22].

Not working and lower level of education were independently associated with poor SRH among thyroid patients. This finding is consistent with other studies [22, 28]. Those who were previously employed had higher odds of poor SRH compared to those currently working. Research demonstrates a reciprocal relationship between unemployment and wellbeing: while unemployment reduces wellbeing, poor wellbeing can also lead to unemployment [29]. No significant differences were observed between those currently working and those who have never worked in multivariable analysis. This may be attributed to the fact that a majority of our study participants were women. The negative impact of unemployment on wellbeing appears to be stronger for men than for women since familial and societal expectations, as well as gendered occupational norms, contribute to low participation of women in the labour force [29].

The effect of multimorbidity, physical and mental health on SRH is widely recognized [30]. In this study, patients with thyroid disorders who had comorbidities were 2.5 times more likely to rate their health as poor compared to thyroid patients without any comorbidity. Future research could explore various combinations of morbidities alongside thyroid disorders, particularly focusing on their contribution to SRH. Patients having limitations in activities of daily living, instrumental activities of daily living, and depression showed a higher likelihood of reporting poor self-rated health. In adults, both excess hyperthyroidism and hypothyroidism are associated with alterations in intellectual performance, while melancholic depression and dementia are prevalent in severe hypothyroidism [31–35]. Recent studies have also indicated a correlation between higher thyroid function levels and an increased incidence of depressive symptoms among older populations [36–38]. However, the neuropsychiatric impairments accompanying dysfunction of the thyroid typically reverse rapidly upon returning to euthyroid status. Although some studies have found no significant effect of thyroid function variations on measures of frailty, prospective studies have linked higher thyroid function, such as subclinical hyperthyroidism, to increased frailty and related parameters, including decreased functional mobility and physical performance in older patients [36]. It is recommended that healthcare providers pay close attention to patients with thyroid disorders who have other co-morbidities, present with limitations in self-care tasks, or symptoms of depression as they may require additional support and interventions to improve their overall well-being. Furthermore, given the clinical effects of thyroid dysfunction, it is important for healthcare providers to consider thyroid function testing and monitoring as part of routine assessments, especially in older populations. This proactive approach may help identify and address thyroid-related issues early, potentially mitigating the development or progression of complications.

Physical activity demonstrated a protective effect in the current study, with non-active individuals showing higher odds of poor SRH. Literature suggests that while physical exercise may not directly affect thyroid function, it can enhance physical fitness, metabolic health, and mental well-being, ultimately improving self-perceived health [39]. Further research is needed to assess the effectiveness of targeted interventions, specifically focusing on physical activity, to understand their impact on SRH.

Healthcare utilization emerged as a significant determinant of self-rated poor health, with poor SRH higher among those who had sought care in the past year. This could be attributed to the immediate need for healthcare prompted by illness or poor health [40, 41]. This is further supported by our study’s finding that among thyroid disorder patients who did not utilize healthcare, around two-thirds cited “did not get sick” or “illness not serious” as reasons.

### Limitations

Since LASI aggregates thyroid conditions under the broad category of ‘thyroid disorders,’ the current study could not analyse which specific type of thyroid disorder is associated with poor SRH. Literature suggests significant physical, emotional, and social impacts of thyroid cancer on HRQoL [42], further affected by treatment and surgical choices. Hypothyroidism has been associated with reduced HRQoL, particularly in areas such as sleep and social isolation, regardless of TPO-Ab levels [43]. Moreover, significant differences in HRQoL were reported between rural and urban patients with Graves’ disease [44]. Evidence of non-toxic goitre on HRQoL remains scarce [45]. The self-reported nature of health outcomes may have also influenced the findings. Although multivariable regression was used to adjust for confounding factors, interactions between gender and presence of comorbidities was not explored which may have influenced the results. Future research should prioritize collecting data on specific thyroid disorders to better understand their distinct impacts, so that tailored interventions could be developed. Future studies should furthermore explore the interactions between gender and comorbidities to gain insights into the combined influence on SRH among those with thyroid disorders.

## Conclusion

The current study revealed that one in four individuals with thyroid disorders perceives their health as poor, and highlighted significant associations of poor SRH with age, residence, caste, religion, employment status, comorbidity, education, physical activity, limitations in ADL, limitations in IADL, depression, and healthcare utilization. Interventions focusing on healthcare access, physical activity, and mental health support are crucial for improving SRH. Routine thyroid function testing, especially in older individuals, is essential for early detection and management of thyroid-related issues. Policymakers and healthcare providers should prioritize holistic healthcare approaches to address the varying needs of individuals with thyroid disorders.

## Data Availability

No datasets were generated or analysed during the current study.
